# Two new species in the genus *Kuvera* Distant, 1906 (Hemiptera, Cixiidae, Cixiinae) from China

**DOI:** 10.3897/zookeys.832.30301

**Published:** 2019-03-20

**Authors:** Yang Luo, Jing-Jie Liu, Ji-Nian Feng

**Affiliations:** 1 Key Laboratory of Plant Protection Resources and Pest Management of the Ministry of Education, Entomological Museum, Northwest A&F University, Yangling, Shaanxi, 712100, China Northwest A&F University Yangling China

**Keywords:** Auchenorrhyncha, Fulgoroidea, morphology, new species, planthopper, taxonomy

## Abstract

Two new species (*Kuverahuoditangensis***sp. n.** and *Kuveralongwangshanensis***sp. n.**) in the family Cixiidae from China are described and illustrated. The generic characteristics are redefined. A checklist to all species of *Kuvera* worldwide and an identification key to the Chinese species are provided. A map of the geographic distribution of *Kuvera* species is also provided.

## Introduction

Cixiidae is the largest family of planthoppers in the world (slightly larger than the Delphacidae), with nearly 2500 described species (Bourgoin 2018). Some cixiids are economically important pests that feed on crops and vector plant pathogens such as: *Hyalesthesobsoletus* Signoret, 1865, Reptalus (Proreptalus) quinquecostatus (Dufour, 1833) and *Myndustaffini* Bonfild, 1983, causing serious economic losses (Julia 1982; Sforza et al. 2010; Pinzauti et al. 2010). Even though this family is very large and important, Cixiidae from the Oriental Region has not been studied extensively.

The genus *Kuvera* Distant,1906 is a member of the tribe Semonini of the subfamily Cixiinae (Hemiptera: Cixiidae). Semonini are characterized by a swollen postclypeus, a convex clypeofrontal suture, and the median carina of frons is incomplete or obscure (Holzinger et al. 2002). Currently, this genus contains 21 species worldwide (Bourgoin 2018). Members of this genus are distributed in China, Korea, Japan, Russia, India, Myanmar and Afghanistan (Distant 1906; Matsumura 1914; Tsaur et al. 1991; Lee and Kwon 1977; Anufriev 2009; Rahman et al. 2017). Previously, nine species in this genus have been recorded from China with eight of them occurring in Taiwan (Matsumura 1914; Tsaur et al. 1991). The Chinese species include: *K.communis* (Tsaur & Hsu, 1991), *K.hama* (Tsaur & Hsu, 1991), *K.laticeps* (Metcalf, 1936), *K.longipennis* (Matsumura, 1914), *K.similis* (Tsaur & Hsu, 1991), *K.taiwana* (Tsaur & Hsu, 1991), *K.tappanella* (Matsumura, 1914), *K.toroensis* (Matsumura, 1914) and *K.transversa* (Tsaur & Hsu, 1991). Since the 1991 study, no further taxonomic work has been done on the genus *Kuvera* in China.

In this paper, we describe and illustrate two new Chinese species of the genus *Kuvera*: *K.huoditangensis* sp. n. and *K.longwangshanensis* sp. n., and we found *K.vilbastei* Anufriev, 1987 for the first time in Tibet, China. We also have provided an amended genus description. A checklist to all worldwide species of *Kuvera* is provided as well as a map of their geographic distribution. We also have developed a key for the Chinese species of *Kuvera*. Differences between *K.flaviceps* (Matsumura, 1900) and *K.longwangshanensis* sp. n. are briefly described.

## Materials and methods

All materials, including holotypes of the new species, were deposited in the Entomological Museum of Northwest A&F University (NWAFU), Yangling, Shaanxi Province, China. Most of their geographical distribution data is based on the localities recorded in the literature, and the rest of the data is based on the collection localities of the specimens examined, which are deposited in Entomological Museum of NWAFU. The updated distribution data is presented in the checklist and on the map.

The morphological terminology and measurements follow Bourgoin et al. (2015) for the venation patterns of the tegmen and Tsaur et al. (1988), Löcker et al. (2006) and Bourgoin (1993) for male and female genitalia.

Measurements of external body length are the distance between the apex of the vertex to the tip of the forewing. Measurements of the vertex length are the distance between the apical transverse carina to the most caudal limits of the vertex.

External morphology was observed using a light LEICA Zoom 2000 microscope. To prepare male genitalia for dissection, specimens were softened for 12h in a humid glass cylinder. The genital segments of specimens were then dissected and macerated in hot 10% NaOH solution overnight or by boiling for 3 to 5 min. The genital segments were then rinsed in distilled water and transferred into PVC microvials containing glycerol. Tissues were immersed in glycerin on slides for drawing. The anal segment and pygofer were drawn. Images were made using a LEICA MZ12.5 stereoscope fitted with a drawing tube and mirror. Photographs of specimens were taken with a Scientific Digital micrography system equipped with an Auto-montage imaging system and a QIMAGING 4000R digital camera (CCD) and imported into Adobe Photoshop CC for labeling and plate composition.

## Taxonomy

### Family Cixiidae Spinola, 1839

#### Subfamily Cixiinae Spinola, 1839

##### Tribe Semonini Emeljanov, 2002

###### Genus Kuvera

Taxon classificationAnimaliaHemipteraCixiinae

Distant, 1906

Kuvera Distant, 1906: 261.

####### Type species.

*Kuverasemihyalina* Distant, 1906.

####### Diagnosis.

**Figures 1–4.**
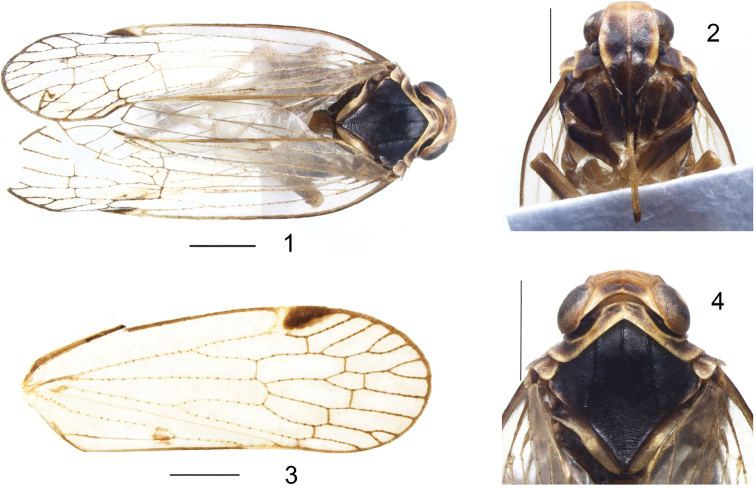
*K.huoditangensis* sp. n. **1**, habitus, dorsal view; **2** frons and clypeus; **3** forewing; **4** head and thorax. Scale bars: 1mm.

Total length varies from 4.7–7.3mm. Body coloration black to yellowish brown. Head including eyes narrower than pronotum. Vertex brown with yellow carinae and borders. Vertex short, wider than long, anterior margin of vertex obscure, with only residual traces. Vertex narrowest at subapical carina, widening towards anterior and posterior margins. Anterior and posterior margins wide and parabolic, almost parallel (Figs 1, 4, 23, 26). Frons prominent, median carina only distinct on basal portion, not reaching the anterior margin of vertex. Both sides of frons usually with yellow to brown stripe, lateral carina slightly elevated, median ocellus small. Frontoclypeal suture sub-semicircular curved upward. Clypeus swollen, postclypeus with prominent median carina, anteclypeus carina sharp or arcuate. Rostrum just reaching hind coxae, apically black (Figs 2, 24). Pronotum small, tapered with obvious carinae and distinct lateral carinae, strongly incised in middle. Mesonotum with three distinct carinate (Figs 1, 4, 23, 26). Tegmina hyaline to semi-hyaline with small granules, slender and longer than abdomen, tectiform. Forewings with a small irregular, roundish spot on anterior branch of Y-vein. Venation pattern: Scp+R usually forked distad of CuA. RP 3-branched, MP with 4 or 5 terminals, CuA 2 or 3-branched, with 10–11 apical cells (Figs 1, 3, 23, 25). Legs yellow, generally 2–4 tibial lateral spines. Hind tibia with 6 apical spines; chaetotaxy of hind tarsi: 7/ (7–8), 2^nd^ tarsal segment with many platellae.

***Male terminalia.*** Pygofer with a triangular medioventral process (Figs 5, 14, 27, 36). Anal segment with a rounded or concave posterior margin (Figs 7, 16, 29, 38, 46). Aedeagus with 2 spinose processes arising near base of flagellum, and flagellum with 1–2 spinose processes. Periandrium almost flat and widened at base. In ventral view, caudal margin of basal segment of periandrium convex, lateral apical angle with two teeth near distal portion (Figs 13, 19, 32, 41).

***Female terminalia.*** Structurally variable among the included species. Ovipositor elongate, orthopteroid and apically curved upwards. 7^th^ sternite (pre-genital sternite) small. Abdominal 9^th^ tergite with a distinct and elliptic wax plate.

####### Remarks.

This genus is similar to the genus *Betacixius* Matsumura, 1914, but can be separated by the following features: Forewings with a small irregular, roundish spot on the anterior branch of the Y-vein, but in *Betacixius*, forewings with a stripe on the anterior branch of the CuA to the posterior portion of A_2_, and a dark long stripe on the nodal lines; one sharp process at about the mid-length of the aedeagal flagellum, but in *Betacixius*, the apex of the flagellum with a sharp process.

####### Distribution.

China (Tibet, Shaanxi, Sichuan, Zhejiang, Taiwan), Korea, Japan, Russia, India, Myanmar, Afghanistan.

###### Checklist and distributions of the species of *Kuvera* Distant, 1906

*K.amurensis* Anufriev, 1987; Russia (Primorsky Krai).

*K.basarukini* Emeljanov, 1998; Russia (Sakhalin).

*K.brunettii* Muir, 1922; India (Eastern Himalayas: Darjeeling).

*K.brunnea* (Dlabola, 1957); Afghanistan (Hindu Kush).

*K.communis* Tsaur & Hsu, 1991; China (Taiwan).

*K.flaviceps* (Matsumura, 1900); Japan (Chishima Islands, Hokkaido, Honshu, Shikoku, Kyushu, Tsushima Island), Korea, Russia (Kuril: Iturups, Kunashir, Shikotan).

*K.hagilsanensis* Rahman, Kwon & Suh, 2017; Korea (Central, South, Jeju-do).

*K.hallasanensis* Rahman, Kwon & Suh, 2017; Korea (Central, South, Jeju-do).

*K.hama* Tsaur & Hsu, 1991; China (Taiwan).

*K.huoditangensis*, sp. n.; China (Shaanxi).

*K.kurilensis* Anufriev, 1987; Russia (Kuriles: Kunashir).

*K.laticeps* (Metcalf, 1936); China (Sichuan).

*K.ligustri* Matsumura, 1914; Japan (Honshu: Hakone, Shikoku, Kyushu, Tsushima Island), Korea.

*K.longipennis* Matsumura, 1914; China (Taiwan).

*K.longwangshanensis* sp. n.; China (Zhejiang).

*K.pallidula* Matsumura, 1914; Russia (Kuriles: Kunashir, Shikotan), Japan (Hokkaido, Honshu).

*K.semihyalina* Distant, 1906; Myanmar (Ruby Mines), India.

*K.similis* Tsaur & Hsu, 1991; China (Taiwan).

*K.taiwana* Tsaur & Hsu, 1991; China (Taiwan).

*K.tappanella* Matsumura, 1914; China (Taiwan).

*K.toroensis* Matsumura, 1914; China (Taiwan).

*K.transversa* Tsaur & Hsu, 1991; China (Taiwan).

*K.ussuriensis* (Vilbaste, 1968); Russia (Khabarovsk), Japan (Hokkaido), China (Sichuan).

*K.vilbastei* Anufriev, 1987; Russia (Primorsky Krai), China (Tibet).

*K.yecheonensis* Rahman, Kwon & Suh, 2017; Korea (Gyeongsangbuk-do).

###### Key to the known species (males) of *Kuvera* from China

**Table:** 

1	Tegmina with 11 apical cells	**2**
–	Tegmina with 10 apical cells	**6**
2	Vertex about 3 times wider than long (Tsaur et al. 1991: fig. 32)	***K.longipennis* Matsumura, 1914**
–	Vertex more than 3 times as wide as long	**3**
3	Periandrium with 2 spinose processes; left process longer than right process	**4**
–	Periandrium with 2 spinose processes; right process longer than left process; in dorsal view, 2 processes cross near middle of periandrium (Tsaur et al. 1991: fig. 28)	***K.transversa* Tsaur & Hsu, 1991**
4	Left process of periandrium S-shaped curve; right process of periandrium hook-shaped curve	**5**
–	Left process of periandrium curved 60 degrees, directed cephalad at apex; right process of periandrium sickle-shaped; middle portion curved outward (Tsaur et al. 1991: fig. 27)	***K.similis* Tsaur & Hsu, 1991**
5	Apex of left process reaching base of periandrium, flagellum with a small and short spine, reaching apex of sclerotized portion of flagellum (Figs 10, 22)	***K.huoditangensis* , sp. n.**
–	Apex of left process not reaching base of periandrium, flagellum with a stout and long spine, reaching middle of membranous portion of flagellum (Fig. 45)	***K.vilbastei* Anufriev, 1987**
6	Periandrium with 2 unequally long spinose processes	**7**
–	Periandrium with 2 nearly equally long spinose processes, approximately equal to half length of periandrium, left process of periandrium curved outward (Emeljanov 2015: fig. 95)	***K.ussuriensis* (Vilbasate, 1968)**
7	Periandrium with 2 spinose processes; left process longer than right process	**8**
–	Periandrium with 2 spinose processes; right process longer than left process	**10**
8	Left process of periandrium S-shaped curve; right process of periandrium sickle-shaped and curved (Tsaur et al. 1991: fig. 30)	***K.hama* Tsaur & Hsu, 1991**
–	Left process of periandrium not S-shaped curve; right process of periandrium not sickle-shaped and curved	**9**
9	Left process of periandrium curved 60 degrees, only directed cephalad at apex; most portions of right process parallel with shaft, apex slightly curved (Tsaur et al. 1991: fig. 29)	***K.communis* Tsaur & Hsu, 1991**
–	Left process of periandrium gently curved from left side to right side, apex curved over shaft and towards the right side; right process of periandrium, touching shaft apically, apex curved and directed ventrally (Figs 35, 44)	***K.longwangshanensis* , sp. n.**
10	Left process of periandrium not curved across shaft	**11**
–	Left process of periandrium curved across the shaft, apex curved and directed cephalad; right process of periandrium almost straight, directed outward (Anufriev 1987: figs 13–16)	***K.laticeps* (Metcalf, 1936)**
11	Left process of periandrium not S-shaped curve; right process of periandrium slightly semi-orbicularly curved	**12**
–	Left process of periandrium S-shaped curved; right process of periandrium hook-shaped, curved; in dorsal view, 2 processes are close and near middle of periandrium (Anufriev 1987: figs 68–71)	***K.toroensis* Matsumura, 1914**
12	In ventral view, 2 spinose processes of periandrium almost straight (Tsaur et al. 1991: fig. 26)	***K.tappanella* Matsumura, 1914**
–	In ventral view, 2 spinose processes of periandrium slightly arched (Tsaur et al. 1991: fig. 25)	***K.taiwana* Tsaur & Hsu, 1991**

####### Kuvera huoditangensis sp. n.

Taxon classificationAnimaliaHemipteraCixiinae

http://zoobank.org/6C717862-1D4D-4D36-9B30-227CEB9A973C

Figs 1–4 , 5–13 , 14–22

######## Type material.

Holotype: male, **China**: Shaanxi, Ningshan County, Huoditang (33°22'N, 108°33'E), 1400–1500m a.s.l., 21.VI.1985, Lan Liu (NWAFU). Paratypes: 1 male, China, Shaanxi, Ningshan County, Huoditang (33°22'N, 108°33'E), 1500m a.s.l., 15.VI.1985, Lan Liu (NWAFU).

######## Description.

Body length: male 6.7–7.0 mm (n=2), forewing length: male 5.8–6.0 mm (n=2).

***Coloration.*** General color black. Body slightly covered with powdery wax (Fig. 1). Eyes dark brown, ocelli milky white. Antenna and rostrum generally dark brown (Fig. 2). Vertex brown with yellow carinae. Frons dark brown near base with lateral carinae yellow brown to pale brown from latero-basal angles to ends of frontoclypeal suture, clypeus black (Fig. 4). Pronotum shallow brown with darker areas. Mesonotum black with 3 dark brown carinae (Figs 1, 4). Tegmina hyaline with veins yellowish and dark brown granules, pterostigma blackish brown. Forewings with a small irregular, roundish spot on anterior branch of Y-vein (Figs 1, 3). Legs and abdomen yellowish brown.

***Head and thorax.*** Vertex about 3.4 times wider than long. Anterior margin of vertex obscure, with only residual traces, subapical transverse carina parabolic, median carina reaching transverse carinae (Fig. 4). Frons slightly swollen, median carina only distinct on basal portion, frontoclypeal suture strongly arcuate. Middle ocelli present. Clypeus swollen, with a visible median carina. Rostrum, just reaching hind coxae (Fig. 2). Pronotum tapered with obvious carinae and distinct lateral carinae, strongly incised in middle. Meso-notum with 3 distinct con-colorous carinae (Fig. 4). Tegmina slender, venation pattern: Scp+R usually forked distad of CuA. RP 3-branched, MP with 4 terminals: MP_1_, MP_2_, MP_3_, and MP_4_, CuA 3-branched, with 11 apical cells (Figs 1, 3). Legs with 3 tibial lateral spines. Hind tibia with 6 apical spines; chaetotaxy of hind tarsi: 7/8, 2^nd^ tarsal segment with 3 platellae.

**Figures 5–13.**
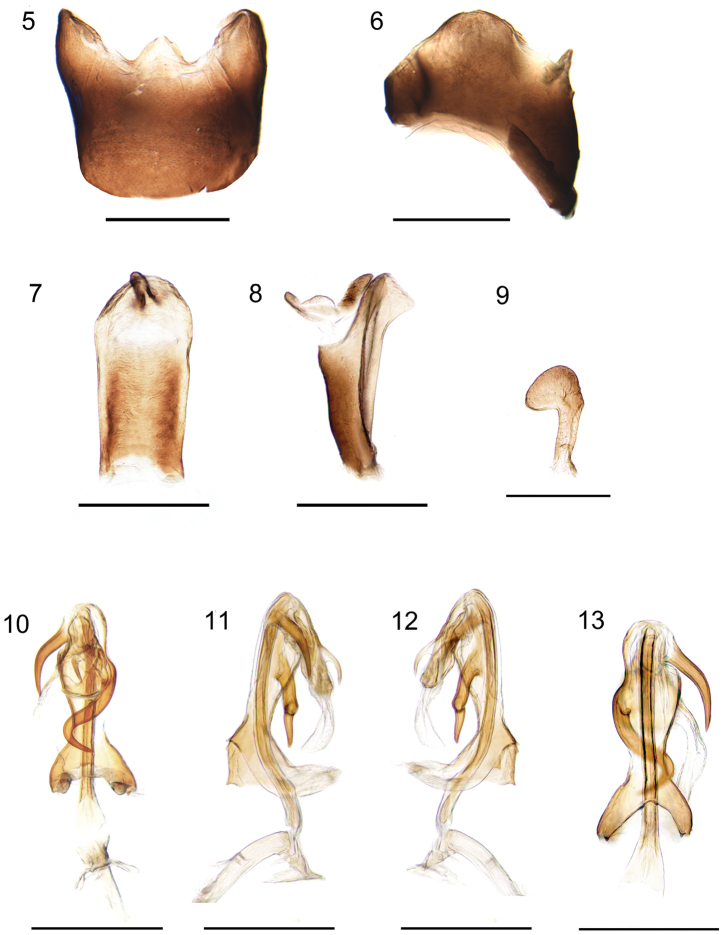
*K.huoditangensis* sp. n. **5** pygofer, ventral view; **6** pygofer, lateral view; **7** anal segment, dorsal view; **8** anal segment, lateral view; **9** genital style, dorsal view; **10** aedeagus, dorsal view; **11** aedeagus, right lateral view; **12** aedeagus, left lateral view; **13** аedeagus, ventral view. Scale bars: 0.5mm.

**Figures 14–22.**
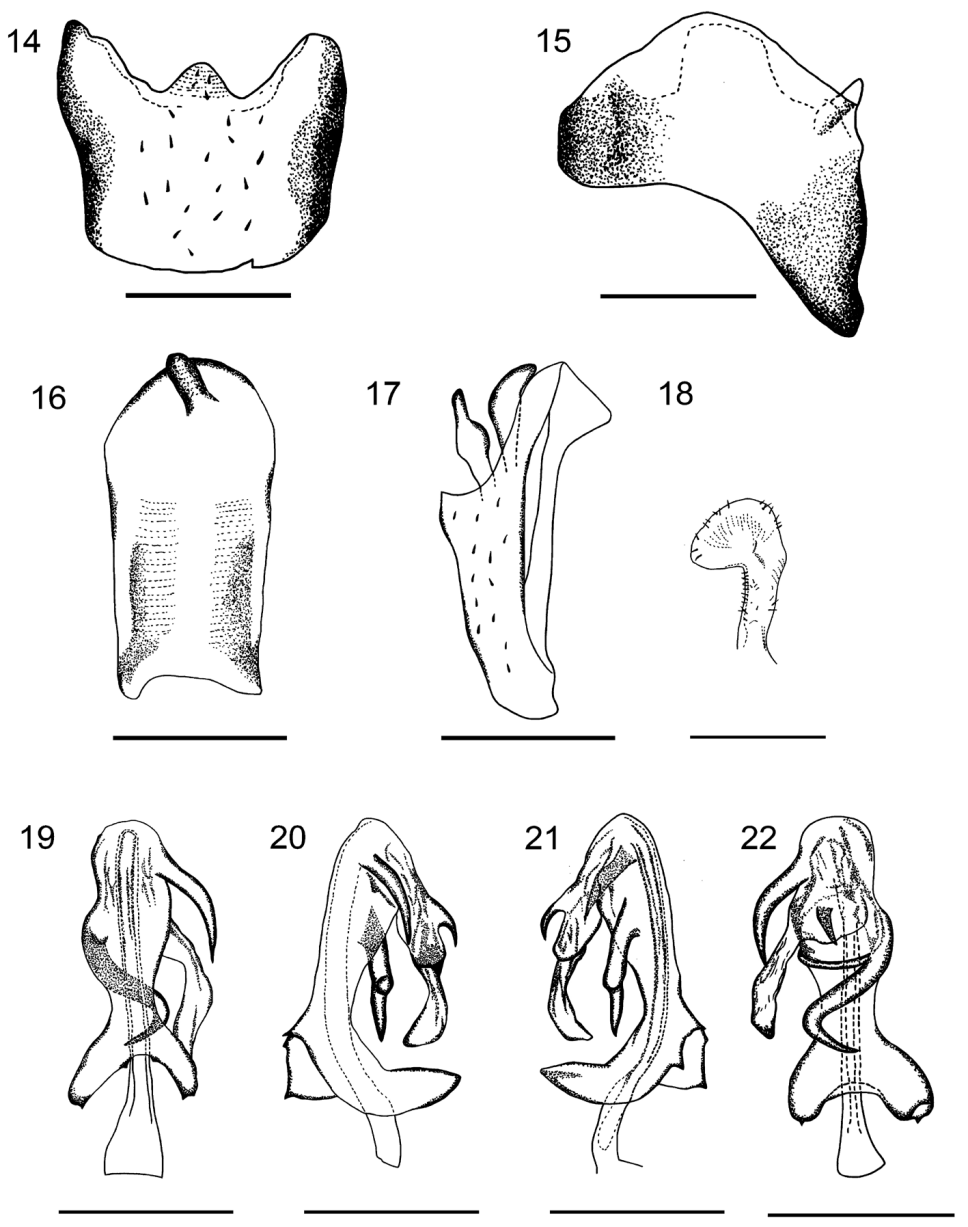
*K.huoditangensis* sp. n. **14** pygofer, ventral view; **15** pygofer, lateral view; **16** аnal segment, dorsal view; **17** anal segment, lateral view; **18** genital style, dorsal view; **19** aedeagus, ventral view; **20** aedeagus, right lateral view; **21** aedeagus, left lateral view; **22** aedeagus, dorsal view. Scale bars: 0.5mm.

***Male terminalia.*** Pygofer with a sub-triangular lateral margin; in dorsal view, asymmetrical, with a triangular medioventral process (Figs 5, 6, 14, 15). Anal segment in lateral view slender, straight at basal part; in dorsal view asymmetrical, longer than broad, widening to middle then narrowing, rounded to apex. Anal style sits subapically (Figs 7, 8, 16, 17). Genital styles symmetrical, in lateral view with hook-shaped apex, parallel-sided at basal half (Figs 9, 18). Aedeagus with 3 spinose processes, in ventral view, periandrium narrow near middle, with 2 spinose processes, one comparatively short, arising near base of flagellum, apex curved and directed cephalad. Another process comparatively long, implanted on left side near mid-length of periandrium, S-shaped, curved from left side to right side and then to middle of periandrium, apex curved 120 degrees and directed left-cephalad (Figs 13, 19). In dorsal view, flagellum with a small and short spine extending from middle, reaching apex of the sclerotized portion of flagellum, directed cephalad. Tip of flagellum near base of periandrium (Figs 10, 22). Periandrium asymmetrically widened at base, slightly curving to left. In ventral view, caudal margin of basal segment of periandrium convex, medially with two teeth, lateral apical angle with two teeth near distal portion (Figs 13, 19).

***Female terminalia.*** Unknown.

######## Etymology.

This species epithet is named after the type locality Huoditang.

######## Distribution.

China (Shaanxi).

######## Remarks.

This new species is similar to *K.vilbastei* but can be separated by the following characteristics: (1) the process implanted on the left side near the mid-length of periandrium (*K.huoditangensis* has a long and S-shaped spinose process, curved from the left side to the right side and then to the middle of the periandrium, apex curved 120 degrees and directed left-cephalad, but *K.vilbastei* has a long spinose process, curved from the left side to the right side, across the shaft sub-apically, apex curved 90 degrees and directed cephalad, not reaching the base of the periandrium); (2) the process extending from the middle of the flagellum (*K.huoditangensis* has a small and short spine, reaching the apex of the sclerotized portion of flagellum, but *K.vilbastei*has a stout and long spine, reaching the middle of the membranous portion of flagellum); and (3) the basal segment of periandrium (*K.huoditangensis* asymmetrically widens in dorsal view, slightly curving to the left, caudal margin of the basal segment of the periandrium convex, medially with two teeth, but *K.vilbastei* symmetrically widens, in ventral view, caudal margin of the basal segment of periandrium convex and serrated).

####### Kuvera longwangshanensis sp. n.

Taxon classificationAnimaliaHemipteraCixiinae

http://zoobank.org/C4EFC153-B71B-4716-97A8-988089BCBEAA

Figs 23–26 , 27–-35 , 36–44

######## Type material.

Holotype: male. **China**: Zhejiang, Anji County, Longwangshan (30°23'N, 119°23'E), 1000–1200m a.s.l., 6/8.VIII.2000, Wu Dai & Cong Wei (NWAFU). Paratypes: 2 males, same data as holotype.

######## Description.

Body length: male 5.1–5.6 mm (n=3), forewing length: male 5.2–5.3 (n=3).

**Figures 23–26.**
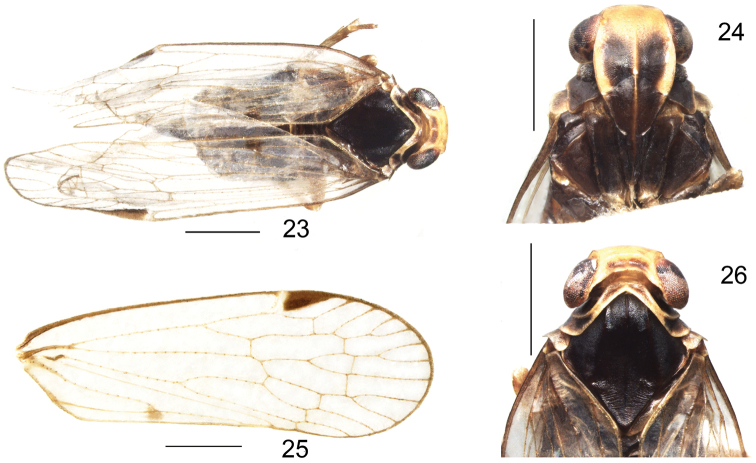
*K.longwangshanensis* sp. n. **23** habitus, dorsal view; **24** frons and clypeus; **25** forewing; **26** head and thorax. Scale bars: 1mm.

***Coloration.*** General color black. Body slightly covered with powdery wax (Fig. 23). Eyes dark brown, ocelli white. Antenna and rostrum generally dark brown (Fig. 24). Vertex brown, apical margin of vertex and surroundings yellow (Fig. 26). Frons dark brown, apical and lateral margins of frons yellowish brown, adjacent area of middle carinae near middle to frontoclypeal suture dark, and V-shaped, frontoclypeal suture and clypeus blackish brown, median carina yellowish (Fig. 24). Pronotum dark brown with yellowish areas. Mesonotum black with 3 dark brown carinae (Figs 23, 26). Tegmina hyaline with veins brown and yellow brown granules, pterostigma blackish brown. Forewings with a small irregular, roundish spot on anterior branch of Y-vein (Figs 23, 25). Legs brown, abdomen dark brown.

***Head and thorax.*** Vertex about 3.8 times wider than long. Lateral and transvers carinae slightly elevated, sub-apical transverse carina parabolic, median carina reaching transverse carinae (Fig. 26). Frons slightly swollen, median carina only distinct on basal portion, frontoclypeal suture strongly arcuate. Middle ocelli present. Clypeus swollen, with a visible median carina. Rostrum, just reaching hind coxae (Fig. 24). Pronotum tapered with obvious carinae and distinct lateral carinae, strongly incised in middle. Mesonotum with 3 distinct con-colorous carinae (Figs 23, 26). Tegmina slender, venation pattern: Scp+R usually forked distad of CuA. RP 3-branched, MP with 5 terminals: MP_11_, MP_12_, MP_2_, MP_3_, and MP_4_, CuA 2-branched, with 10 apical cells (Figs 23, 25). Legs with 3 tibial lateral spines. Hind tibia 6 apical spines; chaetotaxy of hind tarsi: 7/8, 2^nd^ tarsal segment with 4 platellae.

**Figures 27–35.**
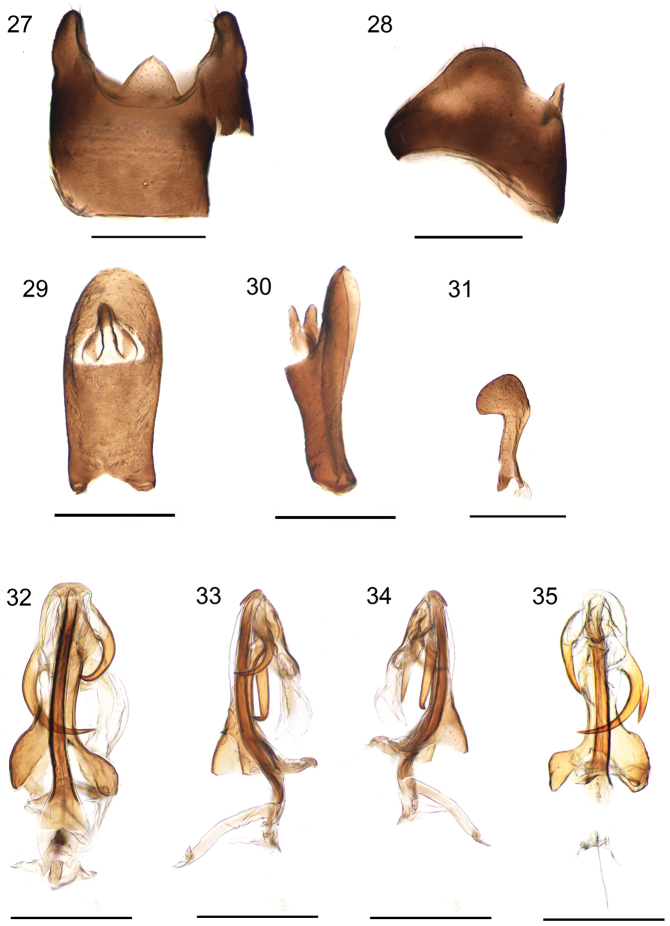
*K.longwangshanensis* sp. n. **27** pygofer, ventral view; **28** pygofer, lateral view; **29** anal segment, dorsal view; **30** anal segment, lateral view; **31** genital style, dorsal view; **32** aedeagus, ventral view; **33** aedeagus, right lateral view; **34** aedeagus, left lateral view; **35** aedeagus, dorsal view. Scale bars: 0.5mm.

**Figures 36–44.**
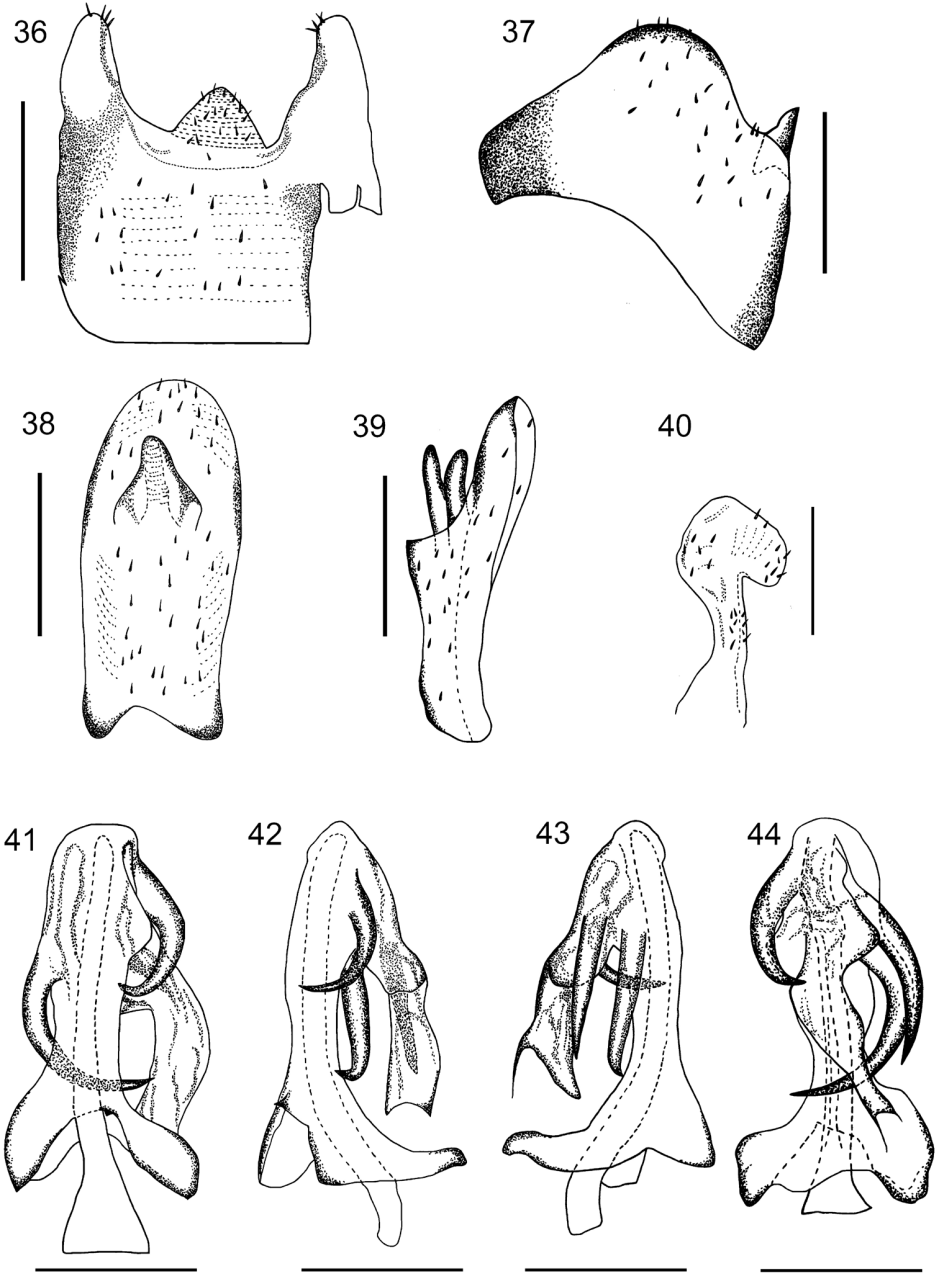
*K.longwangshanensis* sp. n. **36** pygofer, ventral view; **37** pygofer, lateral view; **38** anal segment, dorsal view; **39** anal segment, lateral view; **40** genital style, dorsal view; **41** aedeagus, ventral view; **42** aedeagus, right lateral view; **43** аedeagus, left lateral view; **44** aedeagus, dorsal view. Scale bars: 0.5mm.

***Male terminalia.*** Pygofer with lateral margin sub-triangular in outline; in dorsal view, asymmetrical, wider than long, with a triangular medioventral process (Figs 27, 28, 36, 37). Anal segment in lateral view slender, widening in the middle and then narrowing, rounded at the apex; in dorsal view asymmetrical, longer than broad, narrow near base, expanded sub-apically (Figs 29, 30, 38, 39). Genital styles symmetrical, in lateral view with hook-shaped apex, inner margin deeply concave but outer margin rounded (Figs 31, 40). Aedeagus with 3 spinose processes, in ventral view, periandrium narrow near middle, with 2 spinose processes, the length of shorter spinose process about two-thirds of the longer spinose process. The longer one implanted on the left side near the mid-length of periandrium, gently curved from left to right side, apex curved over shaft and towards the right side. The shorter one arising near base of flagellum, touching shaft apically, apex curved and directed ventrally (Figs 32, 41). In dorsal view, flagellum with a stout and long spine extending nearly one-third the length of flagellum, the length of this spine more than two-thirds that of the longest spinose process, directed cephalad. The tip of flagellum reaches the base of the periandrium (Figs 35, 44). Periandrium asymmetrically widens at base, slightly curving to left. In ventral view, caudal margin of the basal segment of the periandrium convex, medially with a tooth, lateral apical angle with two teeth near the distal portion (Figs 32, 41).

***Female terminalia.*** Unknown.

######## Etymology.

This species epithet is named after the type locality Longwangshan.

######## Distribution.

China (Zhejiang).

######## Remarks.

This new species is similar to *K.flaviceps*, but can be separated by the following characteristics: (1) in dorsal view, the process implanted on the left side near the mid-length of the periandrium (*K.longwangshanensis* has a long spine, gently curved from left side to right side, apex curved over the shaft and to the right side, but *K.flaviceps* has a long spine, gently curved from the left to right side, apex not reaching the right lateral margin of the periandrium); (2) the process arising near the base of the flagellum (*K.longwangshanensis* has a shorter spine, touching the shaft apically, apex strongly curved mesad and directed ventrally, but *K.flaviceps* has a spine not touching the shaft apically, apex slightly curved and directed cephalad); and (3) the process of the flagellum (*K.longwangshanensis* has a stout and long spine extending nearly one-third the length of flagellum, the length of this spine is more than two-thirds of the longest spinose process, directed cephalad; but *K.flaviceps* has a thinner and shorter spine extending from the middle of flagellum, this spine is about half the length of the spinose process).

####### Kuvera vilbastei

Taxon classificationAnimaliaHemipteraCixiinae

Anufriev, 1987

Figs 45–50

Kuvera vilbastei Anufriev, 1987: 7, figs 17–22.

######## Type material.

1 male, China, Tibet Autonomous Region, Bomi Country, Yigong (29°85'N, 95°79'E), 2300m a.s.l, 29.VII.1978, Fa-Sheng Li (NWAFU); 1 male, China, Tibet Autonomous Region, Yadong Country (27°55'N, 88°93'E), 2800m a.s.l, 24.VIII.1978, Fa-Sheng Li (NWAFU).

######## Distribution.

Russia (Primorsk), China (Tibet).

######## Plants associations.

Cedar (*Cedrusdeodara* (*Roxb.*) *G. Don*).

######## Remarks.

**Figures 45–50.**
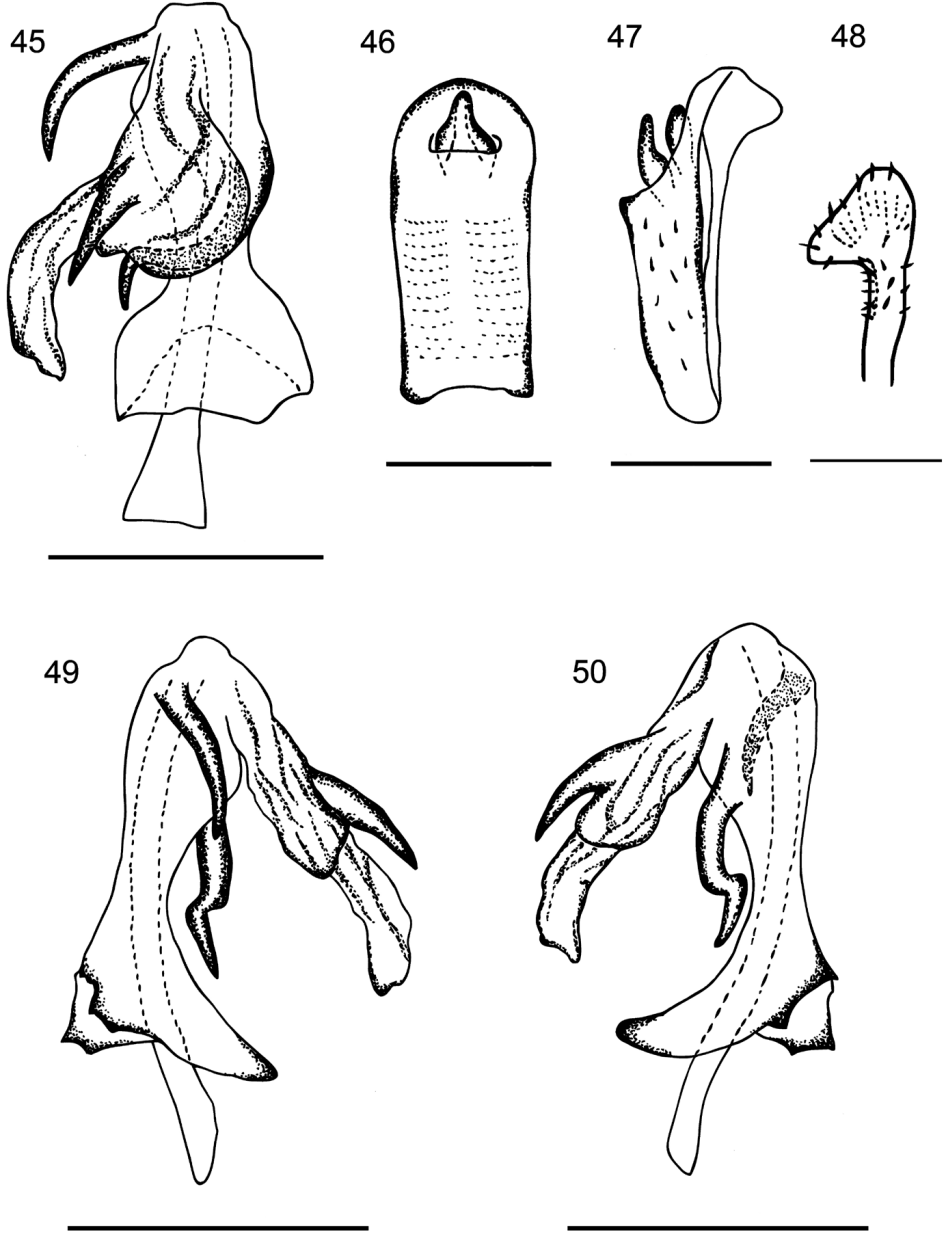
*Kuveravilbastei* Anufriev, 1987. **45** aedeagus, dorsal view; **46** anal segment, dorsal view; **47** аnal segment, lateral view; **48** genital style, dorsal view; **49** aedeagus, right lateral view; **50** aedeagus, left lateral view. Scale bars: 0.5mm.

Based on the description and figures by Anufriev (1987), this species can be distinguished from other species in this genus by following characters: Pygofer with subtriangular lateral margin; with a triangular medioventral process. Anal segment in lateral view slender, straight at basal part; in dorsal view, asymmetrical, about 3 times longer than broad, slightly widening at middle, rounded at apex. Anal style sits subapically (Figs 46, 47). Genital styles symmetrical, in lateral view with hook-shaped apex (Fig. 48). Aedeagus with 3 spinose processes, in dorsal view, periandrium narrow near middle, with 2 spinose processes, one comparatively short, arising near the base of flagellum, apex curved and directed cephalad. Another one comparatively long, implanted on the left side near the mid-length of periandrium, curved from left to right side, curving across the shaft subapically, apex curved 90 degrees and directed cephalad, not reaching the base of the periandrium. Flagellum with a stout and long spine extending from the middle, reaching the middle of the membranous portion of flagellum, directed cephalad. Flagellum reaching the base of the periandrium (Fig. 45). Periandrium symmetrically widened at base, caudal margin of the basal segment of the periandrium convex and serrated, lateral apical angle with two teeth near the distal portion (Figs 49, 50).

####### Kuvera flaviceps

Taxon classificationAnimaliaHemipteraCixiinae

(Matsumura, 1900)

Oliarus flaviceps Matsumura, 1900: 208.
Kuvera flaviceps Matsumura, 1914: 407 (Fig. 2).

######## Distribution.

Japan (Chishima Islands, Hokkaido, Honshu, Shikoku, Kyushu, Tsushima Island), Korea, Russia (Kuril: Iturups, Kunashir, Shikotan).

**Figure 51.**
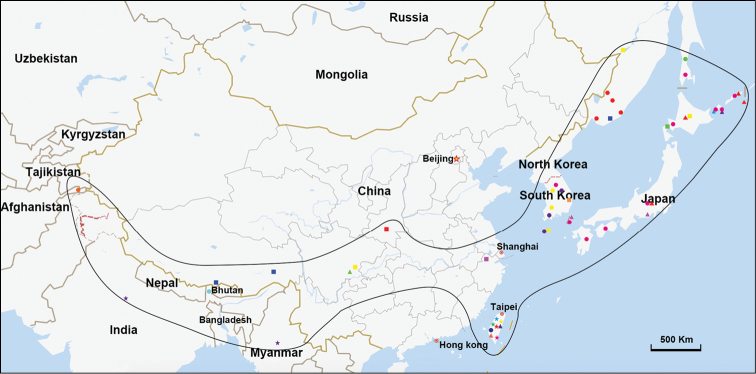
Geographic distribution of *Kuvera* species: *K.amurensis* (⬤); *K.basarukini* (⬤); *K.brunettii* (⬤); *K.brunnea* (⬤); *K.communis* (⬤); *K.flaviceps* (⬤); *K.hagilsanensis* (⬤); *K.hallasanensis* (⬤); *K.hama* (▲); *K.huoditangensis***sp.n.** (■); *K.kurilensis* (▲); *K.laticeps* (▲); *K.ligustri* (▲); *K.longipennis* (▲); *K.longwangshanensis***sp. n.** (■); *K.pallidula* (▲); *K.semihyalina* (★); *K.similis* (★); *K.taiwana* (★); *K.tappanella* (★); *K.toroensis* (★); *K.transversa* (★); *K.ussuriensis* (■); *K.vilbastei* (■); *K.yecheonensis* (■); distribution range of *Kuvera* species (▂).

######## Plants associations.

Birches (*Betulaplatyphylla* Suk.) and hairy alder (*Alnusjaponica* (Thunb.) Steud.).

######## Remarks.

Based on the description and figures by Matsumura (1914) and Anufriev (1987), this species can be distinguished from other species in this genus by the following characters: Pygofer has a lateral margin with a subtriangular outline; in dorsal view, asymmetrical, wider than long, with a triangular medioventral process. Anal segment in lateral view slender; in dorsal view asymmetrical, longer than broad, with convex lateral margins, rounded apically. Genital styles symmetrical, in lateral view with hook-shaped apex. Aedeagus has 3 spinose processes, in dorsal view, periandrium narrows near middle, with 2 spinose processes. A spine is implanted on the left side near the mid-length of the periandrium, which gently curves from the left side to right side, apex not reaching the right lateral margin of the periandrium. Another spine arises near the base of flagellum, not touching the shaft apically, apex curved and directed cephalad. Flagellum with a stout and long spine extending from the middle, this spine is about half as long as the longest spinose process, directed cephalad. Flagellum reaches the base of the periandrium.

## Discussion

The biology of *Kuvera* species throughout the world have not been extensively studied. According to our collection surveys, these species are primarily found on grass, trees, shrubs and forbs, ranging in altitude from 0 to 3000 m a.s.l. The plant associations of *Kuvera* have been described in several previous studies. Anufriev (1987) described cedar and birches as the primary host plants of *K.vilbastei* and *K.pallidula*. Emeljanov (2015) listed the following host plants for *K.ussuriensis* and *K.flaviceps*: myrica (*Myrica L.*), Nanking cherry (*Cerasustomentosa* (Thunb.) Wall.), birches (*Betulaplatyphylla* Suk.) and alder (*lnus japonica* (Thunb.) Steud.). We also found members of this genus on the cedar (*Cedrusdeodara* (Roxb.) G. Don).

As part of ongoing monitoring studies in Chinese agroecosystems, we collected specimens of Cixiidae from crop plants, trees, forbs, shrubs and weeds in locations primarily in Southern China. We found that *K.huoditangensis* sp. n. occurs in Ningshan County, which is on the southern slope in the middle of the Qingling Mountain range. The specimens were collected in Huoditang Teaching and Experimental Forest Farm of Northwest A&F University of Ningshan County at an elevation between 1400 to 1500 m. *Kuveralongwangshanensis* sp. n. occurs in the Longwang Mountain National Nature Reserve (LNNR) of Anji County in the northwest of Zhejiang Province at 200 to 1500 m a.s.l. LNNR is located in the hinterland of the Yangtze River Delta and is covered by virgin forests. In the LNNR, the specimens were collected on Longwang Mountain at an elevation between 1000m and 1200m.

The *Kuvera* genus is distributed in eastern Asia, central Asia and the Indo-Malayan region (Fig. 51). Most *Kuvera* species occur in the Oriental region of the world: China (Sichuan, Zhejiang, Taiwan, south of Qinling Mountain in Shaanxi), India and Myanmar. Some species of *Kuvera* mainly occur in adjacent regions in the northeast Palaearctic such as Primorsk, Khabarovsk, and the Kuriles (eastern Russia); Hokkaido (northern Japan); and the Korean Peninsula. A few species of *Kuvera* occur in the southwest Palaearctic such as the Tibet Autonomous Region (western China) and the Hindu Kush (eastern Afghanistan). We anticipate that additional species of *Kuvera* will be found in countries throughout the primary distribution range of this genus.

## Supplementary Material

XML Treatment for Kuvera

XML Treatment for Kuvera huoditangensis

XML Treatment for Kuvera longwangshanensis

XML Treatment for Kuvera vilbastei

XML Treatment for Kuvera flaviceps
